# Number of drugs in the medication list as an indicator of prescribing quality: a validation study of polypharmacy indicators in older hip fracture patients

**DOI:** 10.1007/s00228-014-1792-9

**Published:** 2015-01-09

**Authors:** Björn Belfrage, Anders Koldestam, Christina Sjöberg, Susanna M. Wallerstedt

**Affiliations:** 1 Närhälsan Dals-Ed Health Center, Dals-Ed, Sweden; 2 Department of Geriatrics, Sahlgrenska University Hospital, Mölndal, Sweden; 3 Department of Clinical Pharmacology, Sahlgrenska University Hospital, SE-413 45 Göteborg, Sweden

**Keywords:** Drug therapy, Health care quality assessment, Polypharmacy

## Abstract

**Purpose:**

Indicators based on the number of drugs in the medication list are sometimes used to reflect quality of drug treatment. This study aimed to evaluate the concurrent validity of such polypharmacy indicators, i.e., their ability to differentiate between appropriate and suboptimal drug treatment.

**Methods:**

In 200 hip fracture patients (≥65 years of age), consecutively recruited to a randomized controlled study in Sahlgrenska University Hospital in 2009, quality of drug treatment at study entry was assessed according to a gold standard as well as to indicators based on the number of drugs in the medication list. As gold standard, two specialist physicians independently assessed and then agreed on the quality for each patient, after initial screening with Screening Tool of Older Persons’ potentially inappropriate Prescriptions (STOPP) and Screening Tool to Alert to Right Treatment (START). Suboptimal drug treatment was defined as ≥1 STOPP/START outcomes assessed as clinically relevant at the individual level.

**Results:**

A total of 141 (71 %) patients had suboptimal drug treatment according to the gold standard. The corresponding figures according to the indicators ≥5 and ≥10 drugs were 149 (75) and 49 (25 %), respectively. The sensitivity for the indicators ≥5 and ≥10 drugs to detect suboptimal drug treatment was 0.86 (95 % confidence interval: 0.80; 0.92) and 0.32 (0.25; 0.40), respectively. The specificity was 0.53 (0.41; 0.65) and 0.93 (0.82; 0.97).

**Conclusions:**

The findings suggest that no polypharmacy indicator could serve as a general indicator of prescribing quality; cut-offs for such indicators need to be chosen according to purpose.

## Introduction

Drug therapy is a common and important tool in modern health care. To improve patient health in the best way, drug treatment needs to be optimized according to the specific patient. However, it is well-known that suboptimal pharmacotherapy is common, such as treatment with inappropriate drugs or dosages, and/or omissions of drugs which the patient would probably benefit from [1, 2].

In order to improve quality of drug treatment, valid indicators of prescribing quality are essential. Such indicators may, for example, be used in clinical practice to identify patients with suboptimal drug treatment for whom the treatment needs to be reconsidered. Furthermore, decision-makers may want to measure quality of healthcare provided. The number of drugs in the medication list at various cut-offs has been used as one type of indicator of prescribing quality, both in the scientific literature [3–8] and by decision-makers [9]. Indeed, polypharmacy is a hot topic of today as the number of drugs per patient is increasing [10, 11], and has been associated with death, visits to the emergency department, gastrointestinal bleedings, and fall accidents [12]. Cut-offs frequently used for polypharmacy indicators are five or ten, that is, five or more drugs as well as ten or more drugs.

However, as far as we are aware, the ability of polypharmacy indicators to reflect the quality of drug treatment has not been evaluated. In fact, although such indicators may be easy to measure, they may also include difficulties. For example, undertreatment may be present in patients with many drugs, and a high number of drugs may be fully appropriate in some patients. Thus, evidence on the concurrent validity is lacking, that is, information on the sensitivity (the proportion of patients with suboptimal treatment according to a gold standard, also identified by an indicator), the specificity (the proportion of patients without suboptimal treatment according to a gold standard, also identified by an indicator), and the predictive value (the proportion of patients correctly characterized by the indicator according to the gold standard).

There is no established gold standard to determine quality of drug treatment. However, a medical assessment is the key step, as all prescribing has to be made after consideration of the individual patient. This may be a challenge, especially in older people. On the one hand, as the burden of disease increases by age, they often qualify for multiple drugs. On the other hand, they are more sensitive to drugs and drug combinations due to aging organ systems [13, 14]. Thus, an approach to a gold standard for quality of drug treatment may be to let specialist physicians assess the medication list in relation to the patient’s medical history. To ascertain that the assessments are made systematically, it may be useful to start from validated and comprehensive screening tools covering both over- and undertreatment such as the Screening Tool of Older Persons’ potentially inappropriate Prescriptions (STOPP) and the Screening Tool to Alert to Right Treatment (START) [1].

The aim of this study was to investigate the concurrent validity of indicators based on the number of drugs in the medication list, that is, the ability of polypharmacy indicators to reflect the quality of drug treatment. Two cut-offs were focused upon (≥5 and ≥10 drugs).

## Methods

In a cohort of 200 hip fracture patients (≥65 years of age), consecutively recruited to a randomized controlled study in the departments of orthopedics, geriatrics, and medicine at Sahlgrenska University Hospital in 2009 [15], quality of drug treatment at study entry (admission to the hospital) was assessed according to a gold standard as well as to polypharmacy indicators based on the number of drugs in the medication list. The study complies with the Declaration of Helsinki, and ethics approval was obtained from the regional ethical review board in Gothenburg.

Gold standard concerning quality of drug treatment was systematically assessed in two steps which aimed to identify inappropriate and missing drugs. Suboptimal drug treatment was defined as ≥1 inappropriate drugs (overtreatment) or ≥1 missing drugs (undertreatment). For a patient without inappropriate/missing drugs, the treatment was considered appropriate.

First, we identified potentially suboptimal drug treatment by the use of STOPP and START, which provide 65 criteria for potentially inappropriate drugs and 22 criteria for potentially missing drugs, respectively [1]. Then, the clinical relevance of identified STOPP and/or START outcomes was assessed at the individual level. An inappropriate drug was defined as a clinically relevant STOPP outcome. Thus, if the expected benefit of the medication was judged to outweigh the potential harm, such as a neuroleptic drug in a patient with schizophrenia, the STOPP outcome was assessed as not clinically relevant, i.e., not representing an inappropriate drug. A missing drug was defined as a clinically relevant START outcome. Thus, if there was a clinical reason not to treat the patient with the drug, such as an adverse drug reaction or a contraindication, the START outcome was assessed as not clinically relevant, i.e., not representing a missing drug. In order to keep a conservative approach to categorizing drugs as inappropriate or missing, we chose to categorize STOPP and START outcomes not possible to assess concerning clinical relevance (e.g., due to missing information) as not clinically relevant.

The assessments were independently performed by one general practitioner (BB) and one geriatrician (AK). They were based on (i) medical records from both hospital and primary care, and (ii) previously collected data including information on risk of falls, cognition, residence, and glomerular filtration rate. The latter, estimated with the Cockcroft-Gault equation, was dichotomized as either ≥50 or <50 ml/min to fit the STOPP and START criteria. In a final consensus discussion, the two specialist physicians reached agreement on all STOPP/START outcomes, and the clinical relevance of these.

### Statistics

All analyses were performed with SPSS (IBM SPSS Statistics for Windows, Version 20.0, Armonk, NY). The Mann-Whitney and the Chi-square tests were used for comparisons of characteristics between patients characterized according to the polypharmacy indicators ≥5 and ≥10 drugs. We used the Spearman rank correlation test to investigate the correlation between the number of drugs in the medication list and the number of inappropriate/missing drugs. Regarding concurrent validity for polypharmacy indicators, we calculated sensitivity and specificity as well as positive and negative predictive value according to cut-offs of the number of drugs in the medication list. Logistic regression was performed to obtain odds ratios (including 95 % confidence intervals) for suboptimal drug treatment, as well as for over- and undertreatment, according to the polypharmacy indicators ≥5 and ≥10 drugs. Adjustments were made for age, sex, cognition (defined as impaired or not), residence (defined as nursing home or not), and multi-dose drug dispensing (a system which has been associated with an extensive medication list and quality of drug treatment) [3, 4, 16]. Kappa statistics was used to assess inter-rater agreement for STOPP/START outcomes.

## Results

The 200 patients studied had a mean age of 84.5 years, ranging from 65 to 98 years, and 133 (67 %) were women. The mean number of drugs in the medication list was 7.2 (standard deviation 3.9, range 0–21). Compared with patients with <5 and <10 drugs in the medication list, patients with ≥5 and ≥10 drugs more often had multi-dose drug dispensing (Table 1). No statistically significant differences were found regarding age, sex, cognition, or residence.

**Table 1:** Characteristics of patients according to the number of drugs in the medication list at two cut-offs (≥5 and ≥10 drugs)

		≥5 Drugs *n* = 149	<5 Drugs *n* = 51	*P* value	≥10 Drugs *n* = 49	<10 Drugs *n* = 151	*P* value
Age	mean ± SD	84.6 ± 6.5	84.2 ± 8.4		84.9 ± 6.5	84.4 ± 7.2	
	median (range)	85 (65–98)	84 (65–97)	0.68	85 (65–96)	85 (65–98)	0.68
Female sex		95 (64)	38 (75)	0.16	33 (67)	100 (66)	0.89
Multi-dose drug dispensing		87 (58)	13 (25)	<0.001	34 (69)	66 (44)	0.002
Impaired cognition		72 (48)	18 (35)	0.11	27 (55)	63 (42)	0.10
Residing in nursing home		47 (32)	13 (25)	0.42	19 (39)	41 (27)	0.12
Number of drugs		8.8 ± 3.0	2.4 ± 1.4	NA	12.2 ± 2.5	5.5 ± 2.6	NA

Values are presented as mean ± SD or number of patients (percentage) if not stated otherwise

*NA* not applicable, *SD* standard deviation

A total of 141 (71 %) patients had suboptimal drug treatment according to the gold standard. The corresponding figures according to the indicators ≥5 and ≥10 drugs were 149 (75) and 49 (25 %), respectively (Table 2). For polypharmacy indicators of ≥1 to ≥10 drugs, the sensitivity ranged between 1.00 (95 % confidence interval: 0.97; 1.00) and 0.32 (0.25; 0.40), and the specificity between 0.14 (0.07; 0.23) and 0.93 (0.82; 0.97). The proportion of individuals with ≥1 to ≥10 drugs confirmed to have suboptimal drug treatment ranged from 73 to 92 % (positive predictive value), whereas 0 to 68 % had suboptimal drug treatment without being identified by these indicators.

**Table 2:** Concurrent validity of indicators of prescribing quality based on cut-offs of number of drugs in the medication list (polypharmacy indicators)

Number of drugs (cut-off for indicator)	Patients identified by the indicator *n*	According to gold standard			Sensitivity (95 % CI)	Specificity (95 % CI)
		Suboptimal drug treatment		Appropriate drug treatment		
		Confirmed *n* (PPV)	Not identified *n* (%)	Identified *n* (NPV)		
≥1	192	141 (0.73)	0 (0)	8 (1.00)	1.00 (0.97; 1.00)	0.14 (0.07; 0.23)
≥2	189	141 (0.75)	0 (0)	11 (1.00)	1.00 (0.97; 1.00)	0.19 (0.10; 0.29)
≥3	176	136 (0.77)	5 (3.5)	19 (0.79)	0.96 (0.93; 0.99)	0.32 (0.22; 0.45)
≥4	163	129 (0.79)	12 (8.5)	25 (0.68)	0.91 (0.86; 0.95)	0.42 (0.30; 0.54)
≥5	149	121 (0.81)	20 (14)	31 (0.61)	0.86 (0.80; 0.92)	0.53 (0.41; 0.65)
≥6	137	111 (0.81)	30 (21)	33 (0.52)	0.79 (0.72; 0.86)	0.56 (0.44; 0.68)
≥7	114	96 (0.84)	45 (32)	41 (0.48)	0.68 (0.61; 0.76)	0.69 (0.57; 0.79)
≥8	84	71 (0.85)	70 (50)	46 (0.40)	0.50 (0.42; 0.60)	0.78 (0.66; 0.86)
≥9	71	61 (0.86)	80 (57)	49 (0.38)	0.43 (0.35; 0.52)	0.83 (0.71; 0.90)
≥10	49	45 (0.92)	96 (68)	55 (0.36)	0.32 (0.25; 0.40)	0.93 (0.82; 0.97)

*CI* confidence interval, *NPV* negative predictive value, *PPV* positive predictive value

The adjusted odds for suboptimal drug treatment were 4.63 times greater in patients with ≥5 drugs in the medication list (95 % confidence interval: 2.21; 9.68). The adjusted odds for over- and undertreatment were 5.98 (2.74; 13.0) and 1.51 (0.70; 3.29), respectively. The corresponding figures for patients with ≥10 drugs in the medication list were 5.05 (1.66; 15.4), 3.39 (1.48; 7.75), and 1.29 (0.64; 2.58).

**Fig. 1:**
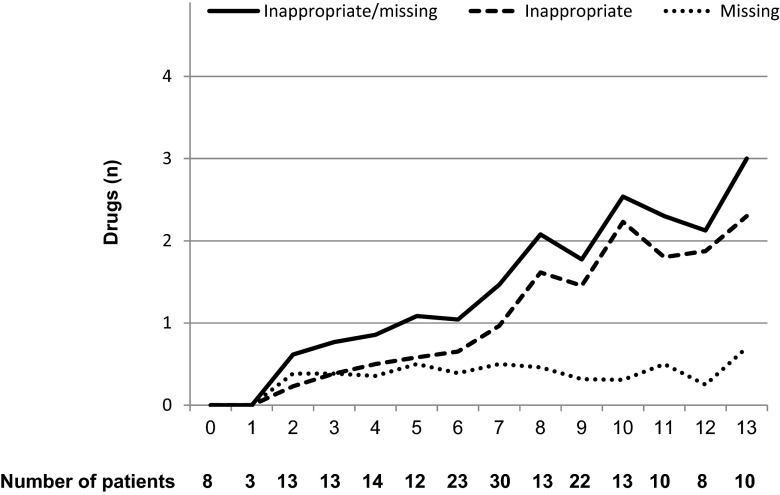
Mean number of inappropriate/missing drugs (y-axis) according to number of drugs in the medication list (x-axis)

In all, 217 inappropriate and 81 missing drugs were identified, the most frequent ones described in Table 3. The inter-rater agreement was moderate (kappa 0.52). The number of inappropriate/missing drugs per patient ranged from 0 to 7, and was positively correlated to the number of drugs in the medication list (*P* < 0.0001; Fig. 1). The most common inappropriate drugs were benzodiazepines, aspirin, loop diuretics, and neuroleptics. Missing drugs included history of cardiovascular disease without beta-blockers, aspirin/clopidogrel, and statins, as well as atrial fibrillation without warfarin.

**Table 3:** Description of the most frequent suboptimal drug treatment, and the prevalence at two cut-offs of number of drugs in the medication list (the polypharmacy indicators ≥5 drugs and ≥10 drugs)

	Type	All *n* = 200	≥5 Drugs *n* = 149	≥10 Drugs *n* = 49
Benzodiazepines in those prone to falls	I	47 (24)	42 (28)	21 (43)
Aspirin at dose >150 mg day	I	23 (12)	20 (13)	7 (14)
Loop diuretic for dependent ankle oedema only, i.e., no clinical signs of heart failure	I	20 (10)	18 (12)	5 (10)
Aspirin with no history of coronary, cerebral or peripheral arterial symptoms or occlusive arterial event	I	13 (6.5)	11 (7.4)	4 (8.2)
Long-term long-acting benzodiazepines	I	13 (6.5)	12 (8.1)	6 (12)
Neuroleptic drugs in those prone to falls	I	11 (5.5)	11 (7.4)	5 (10)
Beta-blocker with chronic stable angina	M	10 (5.0)	9 (6.0)	4 (8.2)
Vasodilator drugs known to cause hypotension in those with persistent postural hypotension	I	9 (4.5)	9 (6.0)	1 (2.0)
Long-term opiates in those with recurrent falls	I	9 (4.5)	9 (6.0)	2 (4.1)
Aspirin or clopidogrel with a documented history of atherosclerotic coronary, cerebral or peripheral vascular disease in patients with sinus rhythm	M	9 (4.5)	8 (5.4)	1 (2.0)
Statin therapy with a documented history of coronary, cerebral or peripheral vascular disease, where the patient’s functional status remains independent for activities of daily living and life expectancy is >5 years	M	9 (4.5)	7 (4.7)	2 (4.1)
Prolonged use of first generation antihistamines	I	7 (3.5)	7 (4.7)	4 (8.2)
Warfarin in the presence of chronic atrial fibrillation	M	7 (3.5)	7 (4.7)	1 (2.0)
Oestrogens without progestogen in patients with intact uterus	I	6 (3.0)	6 (4.0)	4 (8.2)

Values are presented as number of patients (percentage)

*ACE* angiotensin converting enzyme, *I* inappropriate drug, *M* missing drug

## Discussion

### Main study findings

Although we found a strong correlation between the number of drugs in the medication list and suboptimal drug treatment, it was not possible to identify a general cut-off for an indicator of prescribing quality. Indeed, sensitivity and specificity varied greatly and inversely according to the number of drugs in the medication list. Therefore, we suggest that cut-offs for polypharmacy indicators should be chosen by purpose as elaborated upon below.

For an indicator of prescribing quality used in clinical practice, a high sensitivity and a high specificity is desirable. Indeed, if the sensitivity is too low, too many patients will be missed for whom the drug treatment needs to be reconsidered. If, on the other hand, the specificity is too low, resources will be spent on activities, e.g., medication reviews, not needed. Our results show that the sensitivity rapidly declines with higher cut-offs for polypharmacy indicators. In fact, at a cut-off of ≥5 drugs in the medication list, 14 % of the patients with suboptimal treatment would not be identified. By comparison, at a cut-off of ≥10 drugs, 68 % of patients in need for drug treatment optimization would be missed. Correspondingly, the specificity increases according to the number of drugs in the medication list. Thus, using a cut-off of ≥5 or ≥10 drugs would imply that 47 or 7 % of the patients identified, respectively, would not have suboptimal drug treatment.

For decision-makers, on the other hand, an indicator of prescribing quality should preferably have a high predictive value. Indeed, trust-worthy indicators are essential for meaningful benchmarking. Furthermore, as pay-for-performance is increasingly used worldwide [17], it is important that resource allocation procedures use validated measures. In this study, the positive predictive value for suboptimal drug treatment ranged between 73 and 92 %. Thus, the risk of suboptimal drug treatment was high irrespective of cut-off chosen. This may imply that merely having drug treatment may be an indicator of suboptimal drug treatment. Furthermore, the negative predictive values for the indicators ≥5 and ≥10 drugs in the medication list were 61 and 36 %, respectively. Thus, 39 and 64 % of the patients with <5 and <10 drugs had quality problems regarding their drug treatment. Taken together, our results suggest that indicators of prescribing quality based on the number of drugs in the medication list may not be appropriate for benchmarking purposes.

### Strengths and limitations

In this study, we provide knowledge on the concurrent validity of polypharmacy indicators based on different cut-offs of number of drugs in the medication list, that is, their ability to reflect the quality of drug treatment. As far as we are aware, such information is lacking in the scientific literature. An important strength of our study is the choice of gold standard to characterize the quality of drug treatment. Indeed, our gold standard focuses on the clinical relevance, at the individual level, of the results of validated screening tools for potential over- and undertreatment. Another advantage is that all assessments were performed by two specialist physicians with expertise in the relevant area. Furthermore, these assessments were based on quite extensive data both from the original study [15] and from hospital and primary care. However, the assessors did not meet with or talk to the patients in person. Such an assessment strategy could have provided an even more accurate assessment, although it was not possible with the present patient material. Furthermore, the inter-rater agreement was moderate, illustrating the subjectivity of clinical judgments and the advantage to involve two assessors.

The fact that we have analyzed hip fracture patients implies both strengths and limitations. Indeed, these patients may represent a relevant subgroup of older patients since hip fracture is a common diagnosis in Sweden where every fourth middle-aged woman will sustain a hip fracture during her lifetime, and one out of three hip fracture patients is a man [18]. Furthermore, suboptimal drug treatment is common in this patient group [19]. However, the prevalence of suboptimal drug treatment, especially inappropriate drugs related to fall risk, may differ from that found in a general population of older people, and the results may therefore mainly be applicable to hip fracture patients and frail older patients. Furthermore, a limitation of this study is that the STOPP/START tools, which were used to systemize the specialist assessments, may not capture all kinds of suboptimal drug treatment.

### Relation to other studies

The number of inappropriate drugs increased by the number of drugs in the medication list, whereas the number of missing drugs increased at ≥2 drugs and remained relatively stable thereafter. For patients with five or more medications, similar associations have been shown [20]. Indeed, many drugs in the medication list may indicate an extensive medical history, and may implicate difficulties to choose the optimal treatment strategy.

Suboptimal drug treatment was about five times as common for patients with an extensive medication list at both cut-offs focused upon in the present study, ≥5 and ≥10 drugs. Treatment with inappropriate drugs contributed most prominently to these figures, whereas the confidence limits for missing drugs crossed the line of unity. Previously, undertreatment alone has been shown to be about five times as common in patients with ≥5 drugs in the medication list [21]. The divergence between these figures may be explained by the fact that the latter study used treatment guidelines as gold standard. Our approach, evaluating the clinical relevance of validated screening tools, probably provides more conservative figures concerning undertreatment.

Interestingly, when looking at frequent specific inappropriate and missing drugs, no general pattern could be identified according to the polypharmacy indicators ≥5 and ≥10 drugs. Thus, inappropriate drugs were not consequently more common when a cut-off of ≥10 was used, and missing drugs were not consequently more common when a cut-off of ≥5 was used.

In conclusion, this study shows that no general polypharmacy indicator can be used to differentiate between appropriate and suboptimal drug treatment. For researchers and decision-makers, we provide data which can be useful when to consider what cut-offs to choose regarding number of drugs in the medication list, for example to identify patients at risk of suboptimal drug treatment. Indeed, older people seem to have a high risk of both over- and undertreatment with drugs no matter how many drugs they have.
